# Measuring the Maturity of Integrated Care in Singapore with the SCIROCCO Exchange Tool

**DOI:** 10.5334/ijic.7747

**Published:** 2024-10-04

**Authors:** Angeline Woon Kee Lim, Clive Tan, Jason Chin Huat Yap

**Affiliations:** 1 Saw Swee Hock School of Public Health, National University of Singapore, Singapore; 2 National Healthcare Group, Singapore; 3 Tan Tock Seng Hospital, Singapore

**Keywords:** Singapore, integrated care, health systems, SCIROCCO

## Abstract

**Background:**

How have we progressed and where are the gaps of integrated care in Singapore? Social-health care provision in the context of an ageing population is critical in the city-state’s management of the unprecedented demand as the proportion of seniors with multiple complex medical needs have almost doubled in the past decade.

**Objective:**

This study measures the maturity level of Singapore’s integrated care, identifies key gaps and discusses their implications using the SCIROCCO Exchange tool, an online self-assessment tool consisting of the 12 dimensions necessary for the provision of integrated care.

**Methods:**

A three-step mixed method Delphi study was used to derive expert consensus. Participants across the social-healthcare sector as well as representatives from all three public healthcare delivery networks with at least five years of experience were included. Participants rated each of the twelve dimensions of the SCIROCCO Exchange tool on a six-point ordinal scale and provided justifications for each rating. Criteria from the RAND UCLA appropriateness method and thematic analysis were adopted for the analysis.

**Results:**

All participants completed the study. The study found five dimensions in the “Initial” maturity level and five dimensions in the “Progressing” maturity level. There were two dimensions which were “Uncertain” because of split responses, possibly due to their differing vantage points and conceptualisations of integrated care. The overall medians were plotted on a spider diagram. The absence of a systematic approach for integrated care was the most common subtheme across all dimensions. This is foundational for integrated care as this would enable stakeholders across health and social care to identify with a common goal.

**Implications:**

The findings emphasise the imperative to reshape social-health care delivery by focusing on foundational dimensions (such as structure, governance and citizen empowerment) to enable progress in other dimensions. Following the conclusion of this study, Singapore initiated a primary care reform with the launch of Healthier SG in July 2023. Future research may wish to explore the impact of Healthier SG on maturity of integrated care in Singapore.

## Introduction

### Background

Singapore faces an ageing population with longer life expectancies and lower birth rates. The Global Burden of Disease 2019 study reported that Singaporeans have the longest life expectancy in the world at 84.9 years [1]. Concurrently, fertility rate has been declining over the years, reaching a low of 1.1 in 2020 [2]. These have contributed to Singapore’s rapidly ageing population, with the proportion of citizens aged 65 and above increasing from 10.1% in 2010 to 16.8% in 2020 and projected to reach 23.7% in 2030 [3]. Additionally, the proportion of seniors with complex medical needs (with three or more chronic diseases) nearly doubled in the past decade from 19.8% in 2009 to 37% in 2017 [4].

Significant efforts have been made to improve care coordination between health and social care providers. In 2017, the Ministry of Health (MOH) reorganised the healthcare system into three regional health systems, namely National Healthcare Group, Singapore Health Services, and National University Health System [5]. A year later in 2018, MOH announced its Beyond Healthcare 2020 strategy, which consisted of three major shifts to strengthen the health system: “Beyond Hospital to Community”, “Beyond Healthcare to Health” and “Beyond Quality to Value” [6].

Concurrently, the Ministry of Social and Family Development (MSF) published the Guidelines for Case Master Action Planning [7] in 2019 to facilitate the integration and alignment of care plans across social care agencies, particularly for vulnerable citizens with multiple social and/or medical care needs who require support services from several care providers.

The integration of health and social care has been increasingly emphasised as evidence show that social determinants have a strong impact on health outcomes [8]. In Singapore, health and social care are governed separately by MOH and MSF respectively, which are in turn supported by coordinating agencies such as the Agency for Integrated Care (AIC) and National Council for Social Service (NCSS). Studies by Valaitis et al [9] and Christian et al [10] demonstrated how integration across care settings would advance the Triple Aim, a framework developed by the Institute for Healthcare Improvement to optimise healthcare performance [11].

While the term “integrated care” is widely used and socialised in both the healthcare and social care sectors, conceptualisations vary across different settings, with differences in their taxonomies across the type, process, breadth, degree, and the ecological level of integration [12,13]. Specifically, the objectives for integration, such as horizontal or vertical integration of services, sectoral, person-centred or whole system integration, is known to vary [12,14].

This study defines integrated care as “patient care that is coordinated across professionals, facilities, and support systems; continuous over time and between visits; tailored to the patients’ needs and preferences; and based on shared responsibility between patient and caregivers for optimizing health” [15]. For this to be fulfilled, many elements will need to be integrated, as described in the 9 Pillars for Integrated Care [16] by the International Foundation for Integrated Care.

The implementation of person-centred integrated care in healthcare systems is complex and it involves multiple stakeholders across levels of the ecosystem. Hence, regular engagements with stakeholders are necessary to assess the level of maturity for the advancement of care integration.

This study adopted the SCIROCCO Exchange tool as it incorporates building blocks for an integrated care system and assesses each dimension individually, to identify strengths and gaps requiring improvement to facilitate the scaling up of integrated care. Other assessment frameworks such as the Chronic Care Model [17], Rainbow Model [18] and Project INTEGRATE [19] were considered but the SCIROCCO Exchange tool was selected as it adopts systems perspective for care integration.

### The SCIROCCO Exchange

The Scaling Integrated Care in Context (SCIROCCO) Exchange was developed by the European Union Health Programme to facilitate the exchange of good practices and scale up integrated care [20]. The SCIROCCO Exchange Online Self-Assessment Tool (OSAT) assesses each dimension in the B3-Maturity Model, developed by the B3 Action Group on Integrated Care [21].

**Figure 1:**
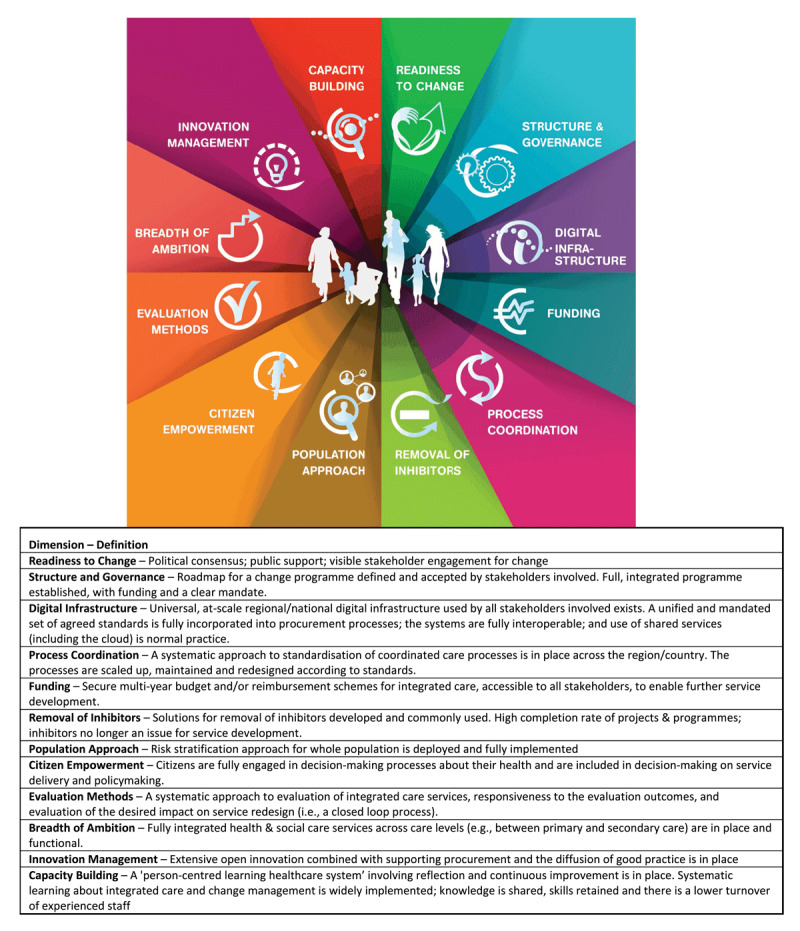
B3-Maturity Model [22].

The B3-Maturity Model (Figure 1) identifies twelve dimensions of integrated care that are necessary in the provision of integrated care [22]. Using the SCIROCCO tool, participants assign a measure of maturity to each dimension of integrated care under the B3-Maturity Model, on a six-point ordinal scale [22]. The tool facilitates an understanding of the maturity of existing social-health care systems in the delivery of integrated care, the identification of strengths and weaknesses for the adoption and transfer of practices, as well as scalability, based on the maturity of each dimension [20].

Validation studies have reviewed the SCIROCCO tool and B3-Maturity Model through a comparison of existing instruments that evaluates the provision of integrated care, with the dimension of the B3-Maturity Model, as well as a Delphi study with experts in integrated care. The studies have found “good internal consistency” [23] and “satisfactory content validity” in the dimensions, maturity indicators and scale of the tool [24]. The SCIROCCO tool has been used in more than 31 organisations, regions and countries, including Scotland, the Basque Country, and the Puglia Region [25].

### Significance of project

Despite ongoing efforts at developing integrated care in Singapore at both the national and regional level, there is a paucity of local literature on Singapore’s progress in integrated care. With insufficient understanding of the maturity of integrated care in Singapore, gaps within the system cannot be adequately identified or addressed.

This research contributes to the understanding of Singapore’s readiness for integrated care, uncovers gaps in dimensions of integrated care, and identifies priorities for the scaling of integrated care. These would facilitate the prioritisation of future efforts and identification of potential foreign models that complement the system’s needs for local adaption.

## Research Methods

### Research Objective and Questions

This study explores Singapore’s maturity of integrated care and readiness for adoption of good practices using the SCIROCCO Exchange online self-assessment tool, seeks to understand the maturity of Singapore’s healthcare system for the provision of integrated care, identifies system strengths and gaps, and explores potential implications of the findings on the future development of integrated care in Singapore.

The research questions are:

1. How mature is integrated care in Singapore as measured with the SCIRROCO tool?
2. In which dimensions of the SCIROCCO model is Singapore progressing well?
3. In which dimensions of the SCIRROCO model are there significant gaps in Singapore?

### The Delphi Process

The Delphi method is an iterative process that systematically derives consensus from a group of experts. In the healthcare setting, it is commonly used when expert opinion is required and where there is limited definitive evidence [26,27,28,29,30,31].

**Figure 2:**
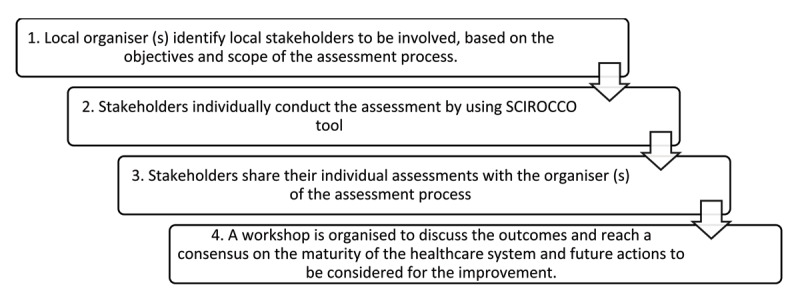
Planning for a Maturity Assessment Process [32].

The self-assessment process in Figure 2 [32] is based on a modified Delphi methodology adopted by regions or countries that utilise the SCIROCCO Exchange tool. An in-person workshop is organised in the second round to facilitate discussions and reach consensus.

The study used the three-step Delphi method developed by Dalkey [33]. According to Cyphert and Grant [34], Brooks [35], Ludwig [36] and Custer, Scarcella, and Steward [37], three iterations are sufficient to achieve expert consensus. The main considerations for the iterative three-step Delphi study were anonymity, expert participants’ time constraints and the evolving COVID-19 pandemic. Participants’ anonymity ensured equal influence and avoided judgement-based bias due to the “bandwagon” and participant dominance effects.

Round 1: Participants were provided with a user guide for the SCIROCCO Exchange tool, which guided them through registration, completion, and submission processes. Participants were asked to rate six statements along an ordinal scale, and provide justifications for their ratings. All responses were then compiled, showing the overall ratings for each dimension on a radar chart. Justifications were summarised and included in the summary report.

Round 2: The compiled report from Round 1 was shared with each, with their own ratings from the previous round. Participants were asked to review their ratings and provide justifications for keeping or amending their ratings.

Round 3: The procedure from Round 2 were repeated in Round 3, which provided a final opportunity for participants to revise their ratings.

### Study Participants

The study targeted experts involved in the design and deployment of health and social care in Singapore. Based on the tool’s recommendation to recruit eight to twelve participants for content validation [38,39], the study recruited twelve industry experts to build this expert consensus.

The study recruited both clinicians and administrators across Singapore’s three regional healthcare clusters, various social service organisations and government agencies. All participants had more than five years of experience in the health and social care sectors. Participants were identified through convenience sampling and invited to participate through electronic mail. All 12 invitees agreed to the study and were provided with an email invitation detailing the study procedures in the participant information sheet. All correspondences were made separately via email to preserve anonymity.

### Data collection

Participants rated each dimension of the SCIROCCO Exchange tool on a six-point scale and provided qualitative justifications for their ratings. Responses for the three Delphi rounds were collected on the SCIORCCO Exchange Knowledge Management Hub (https://scirocco-exchange-tool.inf.ed.ac.uk/en_gb/).

### Data analysis

Criteria from the RAND UCLA appropriateness method [40] was used for data analysis. Responses were categorised into two groups, Agreement and Split, as defined in Table 1. Dimensions that achieved agreement were considered to have derived consensus.

**Table 1:** Distribution of responses.


DISTRIBUTION OF RESPONSES	DEFINITION

Agreement	Less than four ratings outside a 3-point region containing the median

Split	At least four ratings outside the 3-point region containing the median


Options for the six-point scale were categorised into the following maturity levels: “*Initial*”, “*Progressing*”, “*Optimising*” and “*Uncertain*”, based on the median and distribution of responses. The median was preferred to the mean as it is less likely to be influenced by outliers. The operational definitions of levels of maturity are summarised in Table 2.

**Table 2:** Operational definitions of levels of maturity.


MEDIAN	DISTRIBUTION OF RESPONSES	MATURITY LEVEL

0–2	Agreement	Initial

3–4	Agreement	Progressing

5	Agreement	Optimising

Any	Split	Uncertain


Thematic analysis [41] of justifications for each dimension was conducted. Throughout the three rounds, justifications from preceding rounds were retained if the participant retained their ratings in the current round, and were modified or removed when ratings were revised.

## Results

All participants completed three rounds of the study. Participants were grouped into three stakeholder groups based on their role in health and social care, as shown in Table 3.

**Table 3:** Participant Characteristics.


Total number of participants (n)	12

*Background of participants (n, %)*	

Healthcare Administrators	5 (42)

Healthcare Professionals	4 (33)

Social Service Administrators/Professionals	3 (25)

*Years of experience (n, %)*	

5–9	2 (17)

10–14	2 (17)

15 and above	8 (66)


Eight participants submitted all responses for the three rounds through the SCIROCCO Exchange Knowledge Management Hub. Four participants requested to complete the tool on a Microsoft Word or PDF document for at least one round. Their responses were transcribed on the SCIROCCO Hub by the research team.

### Quantitative findings – Ratings

**Figure 3:**
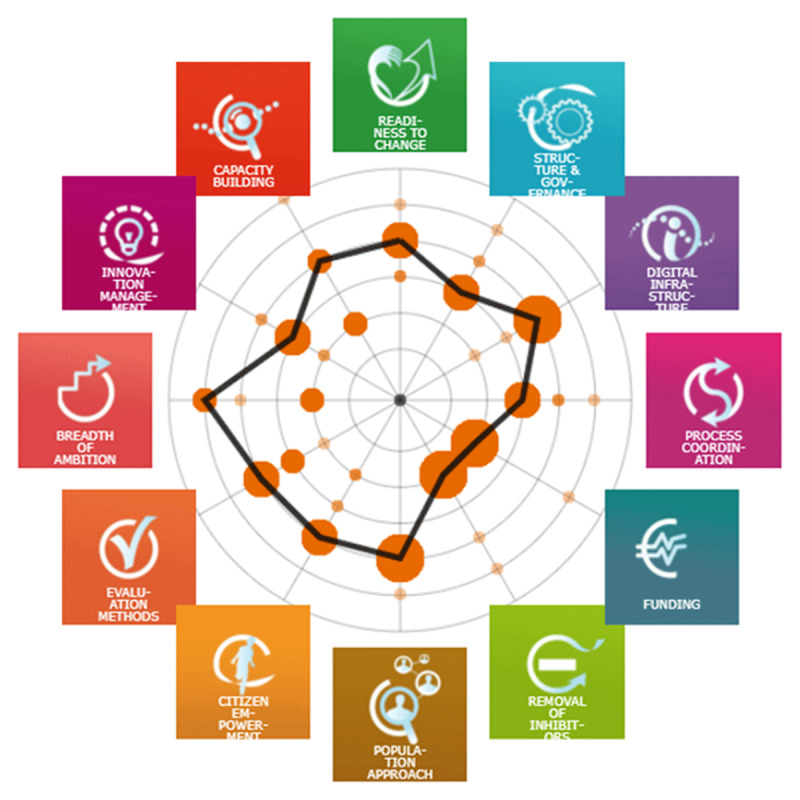
Maturity of Integrated Care based on Overall Median.

The overall study findings are presented using a composite diagram in Figure 3, which indicates that most dimensions saw many participants in agreement on their maturity levels. The definition of ratings for each dimension is included in Appendix 1.

Table 4 below summarises the overall distribution and maturity of integrated care from the three rounds of the study. Overall responses across the three rounds are shown in Appendix 2, while the responses grouped by stakeholder groups over time are in Appendix 3.

**Table 4:** Results of Delphi study based on SCIROCCO tool.


DIMENSIONS	ROUND 1					ROUND 2					ROUND 3				
		
	MEDIAN	% AT MEDIAN	% IN AGREEMENT	DISTRIBUTION OF RESPONSES	MATURITY LEVEL	MEDIAN	% AT MEDIAN	% IN AGREEMENT	DISTRIBUTION OF RESPONSES	MATURITY LEVEL	MEDIAN	% AT MEDIAN	% IN AGREEMENT	DISTRIBUTION OF RESPONSES	MATURITY LEVEL

1 Readiness to change	3.0	66.7	91.7	Agreement	Progressing	3	66.7	100.0	Agreement	Progressing	3	66.7	100.0	Agreement	Progressing

2 Structure & Governance	2.5	37.5	75.0	Agreement	Progressing	2	58.3	91.7	Agreement	Initialising	2	75	91.7	Agreement	Initialising

3 Digital Infrastructure	2.5	20.8	41.7	Split	Uncertain	3	50	83.3	Agreement	Progressing	3	83.3	91.7	Agreement	Progressing

4 Process Coordination	2.5	33.3	66.7	Split	Uncertain	2	58.3	83.3	Agreement	Initialising	2	66.7	91.7	Agreement	Initialising

5 Funding	1.0	58.3	66.7	Split	Uncertain	1	83.3	91.7	Agreement	Initialising	1	91.7	91.7	Agreement	Initialising

6 Removal of Inhibitors	1.0	58.3	66.7	Split	Uncertain	1	75	75.0	Agreement	Initialising	1	83.3	83.3	Agreement	Initialising

7 Population Approach	3.0	50	100.0	Agreement	Progressing	3	83.3	100.0	Agreement	Progressing	3	91.7	100.0	Agreement	Progressing

8 Citizen Empowerment	2.5	33.3	66.7	Split	Uncertain	2.5	37.5	75.0	Agreement	Progressing	3	58.3	75.0	Agreement	Progressing

9 Evaluation Methods	2.5	33.3	66.7	Split	Uncertain	2.5	41.7	83.3	Agreement	Progressing	3	58.3	91.7	Agreement	Progressing

10 Breadth of Ambition	3.0	33.3	66.7	Split	Uncertain	3.5	29.2	58.3	Split	Uncertain	3.5	29.2	58.3	Split	Uncertain

11 Innovation Management	2.0	33.3	83.3	Agreement	Initialising	2	58.3	91.7	Agreement	Initialising	2	66.7	100.0	Agreement	Initialising

12 Capacity Building	3.0	41.7	58.3	Split	Uncertain	3	50	50.0	Split	Uncertain	3	50	50.0	Split	Uncertain


Agreement: Within a 3-point region containing the median.

- **Round 1:** Participants reached agreement on four out of 12 dimensions surveyed. One dimension had “*Initial*” maturity level, three had “*Progressing*” maturity level, and eight had an “*Uncertain*” maturity level.
- **Round 2:** Participants agreed on ten out of 12 dimensions surveyed, six more than in the previous round. Five dimensions had “*Initial*” maturity level, and five had “*Progressing*” maturity level. The number of dimensions with an “*Uncertain*” maturity level decreased from eight to two.
- **Round 3:** In the final round, participants agreed on ten out of 12 dimensions surveyed. Five dimensions had “*Initial*” maturity level, five dimensions were “*Progressing*” and two were ranked “*Uncertain*”. Additionally, there was an increase in percentage of participants with consensus of the group median for dimensions “*Citizen Empowerment*” and “*Evaluation Methods*”. The overall maturity levels of the various dimensions are summarised in Table 5.

**Table 5:** Categorisation of dimensions into maturity levels.


MATURITY LEVELS	DIMENSIONS OF SCIROCCO TOOL

Initial	Structure & Governance, Process Coordination, Funding, Removal of Inhibitors, Innovation Management

Progressing	Readiness to Change, Digital Infrastructure, Population Approach, Citizen Empowerment, Evaluation Methods

Optimising	-

Uncertain	Breadth of Ambition, Capacity Building


### Integrating quantitative and qualitative findings

Triangulation of the quantitative and qualitative findings allowed for exploration of the congruence of findings. In view of the nature of the Delphi study, justifications provided by participants in Rounds 1 and 2 were included in the study if they remained relevant to participants’ ratings for Round 2 and 3 respectively. In instances where participants amended their ratings but retained their comments, these comments were included for analysis. A total of 224 codes were generated from the qualitative data obtained. Two overarching themes were identified from thematic analysis: the progress and the gaps in integrated care, which coincide with this study’s objectives to identify strengths and gaps in the system. Table 6 summarises the subthemes and corresponding dimension/s of the SCIROCCO tool.

**Table 6:** Justifications by participants on ratings of dimensions.


THEMES	SUBTHEMES	DIMENSIONS OF SCIROCCO TOOL	NO. OF DIMENSIONS	CODES (N)	CODES (% OF TOTAL)

**Progress in integrated care**	Citizen’s engagement	Citizen Empowerment	1	4	1.8

	Clear vision or plan with emerging leaders	Readiness to change, Process Coordination	2	7	3.1

	Funding for integrated care initiatives	Readiness to change, Structure and Governance, Funding	3	6	2.7

	Improved coordination in care processes	Readiness to change, Structure and Governance, Process Coordination, Evaluation Methods, Breadth of Ambition, Capacity Building	6	16	7.1

	Innovations captured and mechanisms for knowledge transfer	Innovation Management, Capacity Building	2	10	4.5

	Ongoing dialogue and planning	Readiness to change, Structure and Governance, Removal of Inhibitors, Capacity Building	4	4	1.8

	Risk stratification for specific groups	Population Approach	1	11	4.9

	Structures that enable integrated care	Readiness to Change, Structure and Governance, Digital Infrastructure, Process Coordination, Removal of Inhibitors, Evaluation Methods, Breadth of Ambition, Capacity Building	8	21	9.4

**Gaps in integrated care**	**Absence of systematic approach**	All dimensions	12	34	15.2

	Limited by technical processes	Digital Infrastructure	1	6	2.7

	**Limited coordination and involvement of stakeholders**	Readiness to change, Structure and Governance, Digital Infrastructure, Process Coordination, Funding, Removal of Inhibitors, Population Approach, Breadth of Ambition, Innovation Management, Capacity Building	10	63	28.1

	Limited public awareness and engagement	Readiness to change, Removal of Inhibitors, Citizen Empowerment	3	14	6.3

	**Limited resources and expertise**	Digital Infrastructure, Funding, Removal of Inhibitors, Population Approach, Evaluation Methods, Capacity Building	6	21	9.4

	Policy reform necessary	Removal of Inhibitors, Structure and Governance, Funding, Removal of Inhibitors, Breadth of Ambition	5	7	3.1


The table indicates the number of dimensions and justifications identified for each subtheme. Given that the objective of using the Delphi methodology was to derive a group consensus, identifying the subthemes that a larger proportion of participants related to facilitate the identification of priorities for scaling up of integrated care.

Participants recognised the progress made in integrated care, citing improved coordination in care processes that was however largely limited to pilot programmes or within the healthcare sector. There was acknowledgement of the structures that enabled integrated care, risk stratification for groups at risk of becoming frequent service users, and innovations captured with mechanisms for knowledge transfer.

On the other hand, the limited coordination and involvement of stakeholders, and absence of a systematic approach for integrated care were common subthemes cited across all dimensions, consisting of 28.1% and 15.2% respectively of codes generated. Additionally, the study noted gaps in resources, expertise and policy reform in multiple dimensions.

Table 7 summarises the dimensions of the SCIROCCO tool based on the maturity level, and progress and gaps in care integration. A comparison of spider diagrams of regions across the globe, including the diagram generated from this study, has also been appended in Appendix 4.

**Table 7:** Integration of quantitative and qualitative findings.


DIMENSIONS	OVERALL MEDIAN	CONSENSUS	MATURITY LEVEL	PROGRESS IN CARE INTEGRATION	GAPS IN CARE INTEGRATION

1 Readiness to change	3	Consensus	Progressing	**Clear vision or plan with emerging leaders**, Funding for integrated care initiatives, Improved coordination in care processes, Ongoing dialogue and planning, Structures that enable integrated care	Absence of systematic approach, **Limited coordination and involvement of stakeholders**, Limited public awareness and engagement

2 Structure & Governance	2	Consensus	Initial	Funding for integrated care initiatives, Improved coordination in care processes, Ongoing dialogue and planning, **Structures that enable integrated care**	Absence of systematic approach, **Limited coordination and involvement of stakeholders**, Policy reform necessary

3 Digital Infrastructure	3	Consensus	Progressing	**Structures that enable integrated care**	Absence of systematic approach, Limited by technical processes, **Limited coordination and involvement of stakeholders**, Limited resources and expertise

4 Process Coordination	2	Consensus	Initial	Clear vision or plan with emerging leaders, **Improved coordination in care processes**, Structures that enable integrated care	Absence of systematic approach, **Limited coordination and involvement of stakeholders**

5 Funding	1	Consensus	Initial	**Funding for integrated care initiatives**	Absence of systematic approach, Limited coordination and involvement of stakeholders, **Limited resources and expertise**, Policy reform necessary

6 Removal of Inhibitors	1	Consensus	Initial	Ongoing dialogue and planning, Structures that enable integrated care	Absence of systematic approach, **Limited coordination and involvement of stakeholders**, Limited public awareness and engagement, Limited resources and expertise, Policy reform necessary

7 Population Approach	3	Consensus	Progressing	**Risk stratification for specific groups**	**Absence of systematic approach**, Limited coordination and involvement of stakeholders, Limited resources and expertise

8 Citizen Empowerment	3	Consensus	Progressing	**Citizen’s engagement**	Absence of systematic approach, **Limited public awareness and engagement**

9 Evaluation Methods	3	Consensus	Progressing	Improved coordination in care processes, **Structures that enable integrated care**	Absence of systematic approach, **Limited resources and expertise**

10 Breadth of Ambition	3.5	Split	Uncertain	**Improved coordination in care processes**, Structures that enable integrated care	Absence of systematic approach, **Limited coordination and involvement of stakeholders**, Policy reform necessary

11 Innovation Management	2	Consensus	Initial	**Innovations captured and mechanisms for knowledge transfer**	Absence of systematic approach, **Limited coordination and involvement of stakeholders**

12 Capacity Building	3	Split	Uncertain	Improved coordination in care processes, **Innovations captured and mechanisms for knowledge transfer**, Ongoing dialogue and planning, Structures that enable integrated care	Absence of systematic approach, **Limited coordination and involvement of stakeholders**, Limited resources and expertise


#### Absence of systematic approach for integrated care

Participants suggested a lack of a national, standardised, systematic approach towards health and social care integration, evident in aspects such as funding approaches, digital infrastructure for data sharing, risk stratification and capacity building. While the Beyond Healthcare 2020 [6] vision provided a clear mandate and broad national healthcare strategy, it was far from being operationalised in reality. The “Three Beyonds” provided broad directions but were vague on the ultimate destinations for the health system. Participants felt that there was no national level plan, limited involvement of “smaller players” and limited public awareness of the existing vision for social-health integration. With separate Ministries governing health and social care, policies and priorities might not be aligned, resulting in service duplication and multiple touch points, which limit the potential for social-health integration.

In terms of funding, a healthcare administrator suggested that uncertainties in pilot funding have limited the will and ability of providers to innovate programmes. Participants felt that there needed to be a more systematic and coordinated approach to health and social care funding to improve synergy and cohesiveness of the integration across both sectors.

Participants agreed that risk stratification is not aligned across care settings which affects the scalability of existing approaches. Higher risk groups are prioritised, such as in the Hospital-to-Home (H2H) programme, a transitional care programme targeted at seniors with higher re-admission rates and complex medical conditions [42]. However, not all programmes are designed to be deployed at scale.

In capacity building for integrated care, while there is funding for training across social-health care settings, participants noted the absence of the systematic learning of skills and knowledge for integrated care, with social-health care services remaining in separate silos.

#### Limited coordination and involvement of stakeholders

Participants acknowledged the establishment and existence of structures such as the AIC, RHS and inter-ministry/agency task groups as anchors for leading the system level change and programmatic initiatives for health and social care integration. Participants acknowledged existing infrastructure such as National Electronic Health Records (NEHR) and the AIC’s Integrated Referral Management System (IRMS) facilitate the coordination of care.

For the coordination of social care, the MSF Case Master Action Planning guideline to plan and assign roles of agencies was an example provided. Other standalone initiatives listed by participants include the NUHS Patient Appointment Consolidation programme [43], National One-Rehab framework and National Central Fill Pharmacies to consolidate medication orders across public healthcare institutions [44].

However, participants felt that the progress was limited as existing collaborations between RHS and the community care sector were bilateral point-to-point partnerships and often did not necessarily involve the full range of stakeholders. Participants also felt that smaller stakeholders may also not have a strong influence over national or sub-national population health strategies and developments.

An example provided was that community care staff may not have direct access to an overview of all medical appointments to coordinate transport services for their clients. These limitations surrounding data sharing have hindered the ability of care providers to provide coordinated and holistic care to citizens. Participants were concerned about duplication of services, with many patients accessing multiple health and social care providers.

## Discussion

### A systematic roadmap for change through stakeholder involvement and citizen empowerment are needed to improve coordination of social and health care

This study consolidated the perspectives of health and social sector stakeholders on integrated care, identifying both progresses made over the years and key gaps that remain. The insights from the findings are helpful for health and social care professionals to consider a systematic and strategic approach towards building a stronger coordinated care system for Singapore.

The governance of health and social care in Singapore largely remains distinctly separate under the MOH and MSF. Despite the strategic appointment of the Minister for Social and Family Development as the Second Minister for Health, both ministries maintain distinct budgets and outcome indicators. While participants recognised the role of AIC in facilitating care coordination within the healthcare sector, there is an absence of a national, standardised, systematic approach to health and social care coordination and integration.

A notable example was the Senior Group Homes, an assisted living programme funded by MSF but governed and supported by MOH through AIC [45]. As healthcare clusters play an increasingly significant role in coordinating health and social care, a vision for integrated care is emerging. However, this vision might not be seamlessly streamlined or operationalised across acute and community sectors, leading to potential duplication or service gaps. Additionally, participants acknowledged the bilateral partnerships with some social care organisations that healthcare clusters have developed but most might not have significant influence over national or regional population health strategies.

While participants agreed on most dimensions, there were two dimensions with split responses: breadth of ambition and capacity building. Responses appeared influenced by the participants’ experience and mental models of integrated care, possibly due to their differing vantage points and conceptualisations of integrated care. The split responses for “Breadth of Ambition” and “Capacity Building” indicate the varying interpretations and expectations for integrated care of the participants. This could be attributed to certain developing dimensions, such as “Structure and Governance”, which is in the initial maturity stage but are critical and foundational in setting the stage for a common understanding about integrated care.

A structured and systematic roadmap for change would chart the strategy and identify enablers such as funding, care coordination, digital solutions for data sharing, as examples, to overcome operational challenges because of the separate health and social care ministries. The study found that this is foundational for integrated care and where current gaps are. A systematic roadmap for change would allow stakeholders across health and social care to align their mental models of integrated care and identify with a common goal.

An element of the roadmap for change should also include citizen empowerment and activation, to enable citizens and/or their caregivers to navigate and access health and social care needs. Additionally, citizen consultation is limited due to the lack of a systematic approach and effective policies and the unreadiness of organisations to engage citizens. In general, the perceptions and priorities of citizens for social-health integration are not sought and not known. Thus, further citizen engagement on design of integrated care services and policies is needed to empower citizens to participate in the decision-making process for policies, services and their care.

### Strengths and Limitations

The mixed methods design was adopted to facilitate the triangulation of quantitative ratings assigned and qualitative justifications for the ratings. Though the size of this expert panel reached the recommended range of eight to twelve participants for content validation [38,39], the qualitative nature of the study, group size and composition might have contributed to selection bias, specifically sampling bias and volunteer bias, thus limiting the generalisability of the findings. While the adoption of an online, self-administered tool in the context of the COVID-19 pandemic increased convenience and response rate of the study, there were limited opportunities for participants to clarify their opinions.

### Implications for Public Health

Two key dimensions that would complement a systematic and strategic approach were identified. The first is **structure and governance**. Disparities in the social-health sectors in existing structure, systems and policies were found, due to MOH and MSF’s distinct key performance indicators [46,47] and risk stratification approaches. As a result, citizens end up receiving fragmented care. Through forming a roadmap for change that could be implemented by a joint social-health agency, many other dimensions such as the embodiment of common vision in policies (e.g., readiness to change), process coordination, funding, digital infrastructure, and population approach may also achieve progress.

The second is a shift from healthcare-centric to citizen-centric outcomes through **citizen empowerment**. This is required to better understand citizens’ priorities for care coordination and meet population needs [48]. Porter and Teisberg [49] stated that “*Healthcare is on a collision course with patient’s needs and economic reality*”. As social-health care services remain in the meso- and exo-systems, planning and implementation of integrated care needs to go across the ecosystem and involve individuals. Citizen empowerment would facilitate the understanding of individual needs, for the development of a national strategy that could be systematic yet responsive to various population needs and wants.

When there is a lack of clarity on the macro level, such as structure and governance, stakeholders’ perception of other dimensions may be varied, as shown in the split responses for breadth of ambition and capacity building. Additionally, the progress in other dimensions may be limited. The findings in this paper suggest that there is a sequence to the dimensions of integrated care, where certain dimensions are enablers for progress in other dimensions. A consistent subtheme mentioned by participants is the absence of a systematic approach for integrated care.

## Conclusion

This Delphi mixed methods study offers an understanding of the maturity of integrated care in Singapore with the SCIROCCO Exchange tool. The study found five dimensions in “*Initial*” maturity, five dimensions in “*Progressing*” maturity, and two dimensions with “*Uncertain*” maturity due to split response. The discussion emphasised the need to reshape the way social-health care is delivered by focusing on the foundations, such as structure, governance and citizen empowerment, to enable progress in other dimensions. This requires a Whole of Government approach for a systematic, holistic and coordinated approach for integrated health and social care. Key priorities identified are the governance of social-health agencies and citizens’ consultation on integrated care. Following the conclusion of the study, Singapore has initiated a primary care reform with the launch of Healthier SG in July 2023. Each resident is enrolled to a primary care physician who is accountable for both preventive care and chronic disease management. The primary care physicians would be supported by their team of nurses, coordinators and administrative staff to coordinate the care of residents with community providers. Future research may wish to explore the impact of this transformative effort, which is a progress in dimensions such as structure and governance, on the maturity of other dimensions of integrated care in Singapore.

## Figures and Tables

**Appendix 2:**
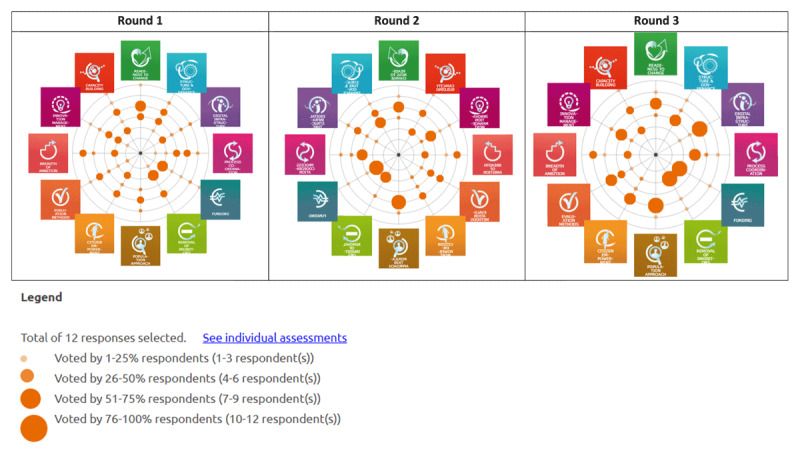
Overall responses of self-assessment tool over time.

**Appendix 3:**
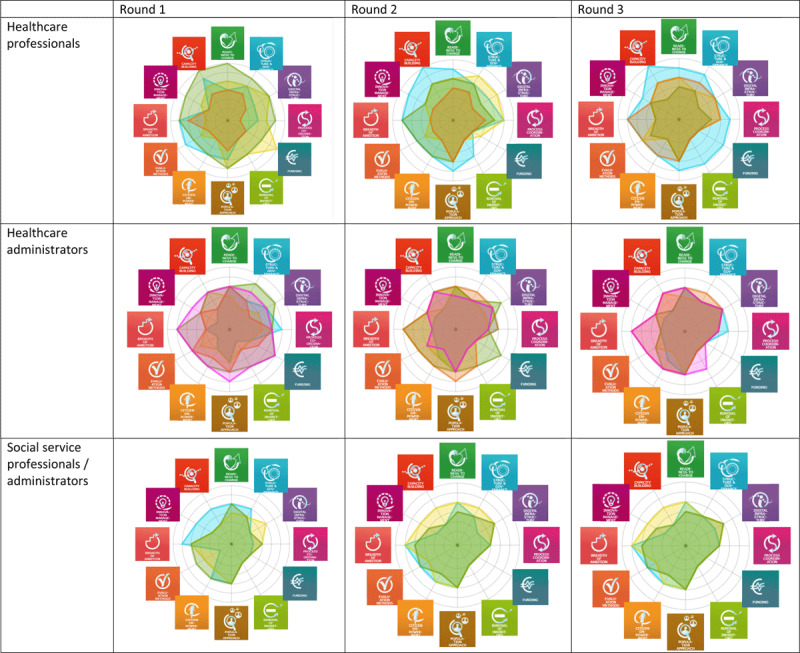
Responses of SCIROCCO tool by stakeholder groups over time.

**Appendix 4:**
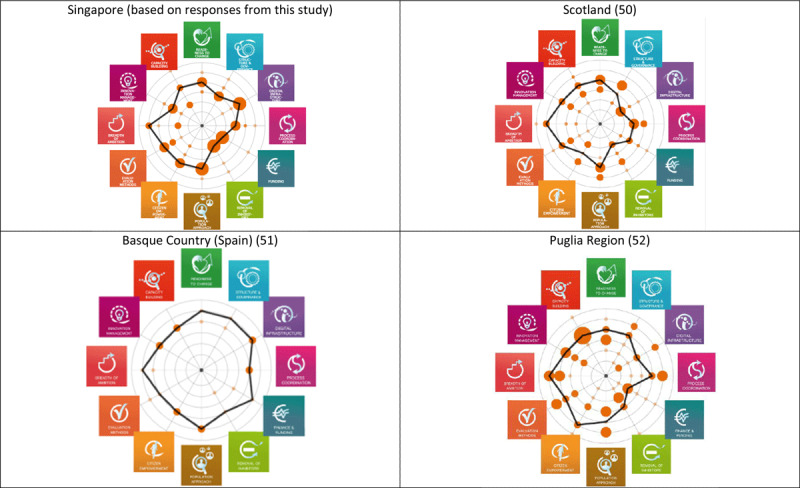
Comparison of SCIROCCO Exchange outcomes across regions.

## Appendix

**Appendix 1:** Descriptions for group rating of 12 dimensions of SCIROCCO tool.

DIMENSION	GROUP MEDIAN	MATURITY LEVEL	DESCRIPTION FOR RATING

Readiness to Change	3	Progressing	*Vision or plan embedded in policy; leaders and champions emerging*

Structure and Governance	2	Initial	*Formation of task forces, alliances and other informal ways of collaborating*

Digital Infrastructure	3	Progressing	*Digital infrastructure to support integrated care are piloted but there is not yet region-wide coverage*

Process Coordination	2	Initial	*Some standardised coordinated care processes are underway; guidelines are used, some initiatives and pathways are formally described, but no systematic approach is planned*

Funding	1	Initial	*Funding is available but mainly for the pilot projects and testing*

Removal of Inhibitors	1	Initial	*Awareness of inhibitors but no systematic approach to their management is in place*

Population Approach	3	Progressing	*Risk stratification used for specific groups, i.e., those who are at risk of becoming frequent service users*

Citizen Empowerment	3	Progressing	*Citizens are consulted on integrated care services and have access to health information and health data*

Evaluation Methods	3	Progressing	*Some integrated care initiatives and services are evaluated as part of a systematic approach*

Breadth of Ambition	1 (5)	Initial	*The citizen of their family may need to act as the integrator of service in an unpredictable way*

	3 (1)	Progressing	*Integration between care levels (e.g., between primary and secondary care) is achieved*

	4 (6)	Progressing	*Improved coordination of social care service and health care service needs is introduced*

Innovation Management	2	Initial	*Innovations are captured and there are some mechanisms in place to encourage knowledge transfer*

Capacity Building	1 (5)	Initial	*Some approaches to capacity building for integrated care services are in place*

	3 (6)	Progressing	*Learning about integrated care and change management is in place but not widely implemented*

	5 (1)	Progressing	*A ‘person-centred learning healthcare system’ involving reflection and continuous improvement is in place*
